# I-BET151 suppresses osteoclast formation and inflammatory cytokines secretion by targetting BRD4 in multiple myeloma

**DOI:** 10.1042/BSR20181245

**Published:** 2019-05-14

**Authors:** Ning-Hong Guo, Ji-Fu Zheng, Fu-Ming Zi, Jing Cheng

**Affiliations:** Department of Hematopathology, The Second Affiliated Hospital of Nanchang University, Nanchang 330006, P.R. China

**Keywords:** BRD4, I-BET151, Multiple myeloma, NF-κB signal pathway, Osteoclast, RANKL

## Abstract

**Background:** Multiple myeloma (MM) is an incurable hematologic cancer, accompanied by excessive osteoclast formation and inflammatory cytokine secretion. The mechanisms by which bromodomain and extra-terminal domain (BET) protein inhibitor I-BET151 regulates osteoclast differentiation and inflammatory cytokine secretion in MM are largely unknown. **Methods**: The isolated peripheral blood mononuclear cells from normal or patients with MM were treated with receptor activator of NF-κB ligand (RANKL) and M-CSF to induce osteoclast differentiation. RAW 264.7 cells were treated with RANKL. I-BET151 was applied to investigate the effects of BRD4 inhibition on osteoclast formation and inflammatory cytokine secretion. Osteoclast formation was determined by tartrate-resistant acid phosphatase (TRACP) staining. The expression of osteoclast-specific genes *TRACP*, matrix metalloproteinase-9 (*MMP-9*), cathepsin K (*Ctsk*), and *c-Src* was tested using quantitative real-time PCR. And the level of inflammatory cytokines TNF-α, IL-1β, and IL-6 was assessed by ELISA. Tumor necrosis factor receptor-associated factor 6 (TRAF6), BRD4, nuclear and cytoplasm p65, IκB-α, nuclear factor of activated T cells cytoplasmic (NFATc1), and osteoprotegerin (OPG) expression were measured by Western blotting. RNAi technology was applied to knock down BET family member BRD4. **Results:** I-BET151 dose-dependently suppressed osteoclast formation, inhibited the levels of osteoclast-specific genes *TRACP, MMP-9, Ctsk*, and *c-Src* and inflammatory cytokines TNF-α, IL-1β, and IL-6 secretion in peripheral blood mononuclear cells and RAW 264.7. I-BET151 inhibited the protein levels of BRD4 and NFATc1, increased OPG expression, and suppressed IκB-α degradation and p65 nuclear translocation. Further, the effects of I-BET151 on osteoclast formation, osteoclast-specific genes expression, inflammatory cytokine secretion, and NF-κB inhibition were promoted by BRD4 knockdown. **Conclusion:** I-BET151 inhibits osteoclast formation and inflammatory cytokine secretion by targetting BRD4-mediated RANKL-NF-κB signal pathway and BRD4 inhibition might be beneficial for MM treatment.

## Introduction

Multiple myeloma (MM) is an incurable cancer developing almost exclusively in the bone marrow and produces focal or diffuse bone destruction [1,2]. It was reported that approximately 30280 new cases of MM and 12590 MM-related deaths occurred in the U.S.A. in 2017 [3]. In the recent decades, MM managements have achieved much progress with the application of bone marrow transplantation together with the combination of proteasome inhibitors and immunomodulatory agents [4]. However, adverse effects such as neutropenia, myelosuppression, thrombocytopenia, and serious infection associated with MM treatment and drug resistance are two major concerns [5]. MM cells generating various inflammatory cytokines, such as TNF-α, IL-6, and IL-1β, and the interaction between myeloma cells and bone microenvironment modulate osteoclast differentiation and osteoblast inhibition [6]. All these contribute to bone destruction and loss, initiating MM development. Alternative targets or therapeutic strategies that regulate osteoclast differentiation and inflammation response could be beneficial for MM treatment.

Receptor activator of NF-κB ligand (RANKL) is essential for osteoclastogenesis [7–9]. Once RANKL binds to its receptor RANK on cell membrane to form RANK/RANKL complex, it recruits tumor necrosis factor receptor-associated factor 6 (TRAF6) followed by the activation of NF-κB signal pathway, resulting into IκB-α degradation and RelA (p65) nuclear translocation [10,11]. This leads to the induction of nuclear factor of activated T cells cytoplasmic (NFATc1), promoting its association with c-Fos and c-Jun [12–14]. The activation of this RANKL-NF-κB signal pathway promotes the osteoclastogenesis and the expression of osteoclast-specific genes, such as tartrate-resistant acid phosphatase (TRACP), matrix metalloproteinase-9 (MMP-9), cathepsin K (Ctsk), carbonic anhydrase 2 (Car2), and integrin β3, which are related to osteoclast differentiation, thereby regulating osteoclast formation [15,16]. The non-canonical NF-κB signal pathway also plays crucial roles in RANKL-mediated osteoclast differentiation [7,17]. For example, TRAF6 influences the development and function of osteoclasts by regulating novel non-canonical NF-κB signaling via B-cell chronic lymphatic leukemia protein 3 (BCL3) deubiquitination in bone marrow-derived macrophages (BMDMs) [7]. Since the non-canonical NF-κB signal pathway regulates telomerase reactivation, it is also possible that telomerase is involved in RANKL-mediated osteoclast differentiation [18]. Administration with osteoprotegerin (OPG) ligand (OGL) into adult mice-induced osteoclast differentiation and activation via directly binding to RANK [19,20]. Polyclonal antibody against the extracellular domain of RANK-stimulated osteoclastogenesis in bone marrow cultures while RANKL knockout mice showed severe osteoclast loss and osteoporosis [19,21]. RANKL-NF-κB signal pathway also mediates inflammation response and inflammatory cytokine secretion [22]. Thus, drugs that target RANKL-NF-κB signal pathway would be beneficial for MM management.

The bromodomain and extra-terminal domain (BET) family, composed of BRD2, BRD3, BRD4, and BRDT, is the most well-known subfamily of eight major BRD families and plays a critical role in the regulation of chromatin modification and genetic transcription [23]. They recognize and bind to the N-acetylation of lysine residues on histone tail, releasing DNA sequences, recruiting transcription factors, and thereby enhancing gene expression [24]. BET proteins could bind to acetylated RelA and coactivate transcription of NF-κB [25]. The inhibition of BET by JQ1 suppressed vascular inflammation by reducing NF-κB activity [26]. And BRD4 was revealed to regulate renal fibrosis by the modulation of NF-κB phosphorylation and acetylation [27]. However, the roles of BRD4 protein in the modulation of osteoclast differentiation via RANKL-NF-κB signal pathway in MM are largely unknown.

I-BET151 is a quinoline class of BET protein inhibitor, showing high selectivity and potency. BET proteins contain acetyl-lysine-binding pockets which could be recognized by acetylated chromatin, mediating gene transcription [26]. I-BET151 competitively binds to the acetyl-lysine-binding pocket in BET proteins, inhibits the recruitment of BET proteins to the acetylated histone tail of its target genes, such as NF-κB [26]. The protective effects of I-BET151 in renal disorders and various cancers, including lung cancer, prostate cancer, and breast cancer, were reported [27–30]. However, whether I-BET151 regulates MM via targetting BRD4 to modulate RANKL-NF-κB signal pathway has not been demonstrated.

The present study for the first time presented that I-BET151 suppressed osteoclast differentiation and inflammatory cytokine secretion by reducing BRD4-mediated NF-κB activation to relieve MM. These may provide an alternative target for the discovery of effective therapeutic drugs for MM treatment.

## Materials and methods

### Cells and reagents

RAW 264.7 cells were purchased from American Type Culture Collection (ATCC, Manassas, VA, U.S.A.). Cell culture medium and Lipofectamine 2000 were from Gibco (Life Technologies, Grand Island, NY, U.S.A.). M-CSF and RANKL recombinant protein factors, FBS, and ELISA kits for TNF-α, IL-1β, and IL-6 were purchased from Thermo Fisher Scientific (Waltham, MA, U.S.A.). I-BET151 was obtained from Santa Cruz Biotechnology (Dallas, Texas, U.S.A.). TRAF6 antibody was purchased from Epitomics, Inc. (Burlingame, CA, U.S.A.) and others including anti-BRD4, anti-p65, anti-IκB-α, anti-NFATc1, anti-OPG, anti-lamin B, and anti-β-actin were all from Cell Signaling Technology (Danvers, MA, U.S.A.). TRACP staining kit was obtained from Nanjing Jiangcheng Bioengineering Institute (Nanjing, China). Non-silencing control siRNA was purchased from QIAGEN (Germantown, MD, U.S.A.) and siRNA targetting BRD4 was synthesized by GenePharma (Shanghai, China). Random hexamer primer and MMLV Reverse Transcriptase were obtained from Promega (Madison, WI, U.S.A.). HotStart SYBR Green qPCR Master Mix (2×) was from TransGen Biotech (Beijing, China). Nitrocellulose membranes were purchased from Millipore (Darmstadt, Germany). Nuclear and Cytoplasmic Protein Extraction Kit was obtained from Beyotime (Jiangsu, China). Other reagents and kits used in the present study were obtained from Sigma (St. Louis, MO, U.S.A.).

### The isolation of peripheral blood mononuclear cells

Human peripheral blood samples were collected from 20 healthy donors and 20 MM patients with their informed consent from The Second Affiliated Hospital of Nanchang University (Nanchang, Jiangxi, China). It was officially approved by Institutional Ethics Research Committee. Human peripheral blood mononuclear cells were isolated using a density gradient technique. Blood mixed with PBS was added on top of the Ficoll–Paque solution followed by centrifugation at 400×***g*** for 35 min at room temperature (RT). The peripheral blood mononuclear cells beyond the Ficoll–Paque were carefully transferred to a new tube. Then they were washed with PBS and centrifuged at 300×***g*** at 4°C twice.

### Cell culture and osteoclasts differentiation induction

The isolated peripheral blood mononuclear cells were incubated with 25 ng/ml M-CSF and 20 ng/ml RANKL in α-MEM supplemented with 10% FBS for 21 days to induce osteoclast differentiation [31], either with or without I-BET151 at indicated concentrations (25, 50, and 100 nM). The medium was replaced every 2–3 days. RAW 264.7 cells were cultured in Roswell Park Memorial Institute (RPMI) 1640 medium supplemented with 10% FBS and 1% penicillin–streptomycin. To induce osteoclast formation, RAW 264.7 cells were incubated with RANKL at 100 ng/ml in the presence or absence of I-BET151 at various concentrations (50, 100, and 200 nM) for 7 days. The medium was replaced every 2–3 days.

### TRACP staining

After required treatment, cells were fixed with 3% paraformaldehyde and 2% sucrose in PBS for 10 min at RT followed by staining for TRACP using a TRACP stain kit according to manufacturer’s protocol. TRACP positive multinucleated cells (≥three nuclei) were considered as differentiated osteoclast-like cells and counted under eight fields of view for each sample and monitored using a Zeiss Axio Observer D1 microscope with ×100 magnification. The images were captured and analyzed with Zeiss ZEN software 2012 (Zeiss GmbH, Jena, Germany).

### ELISA

The cell culture supernatants were collected by centrifugation and subjected to the measurement of TNF-α, IL-1β, and IL-6 protein level ausing ELISA kits following the manufacturer’s instructions. All the measurements were performed in triplicate, and the plates were analyzed with a microplate reader (Synergy NEO, BioTek).

### BRD4 knockdown

The sequences are as below: (Fw, 5′-GGAGAUGACAUAGUCUUAATT-3′, Rv, 5′-GCACAAUCAAGUCUAAACUTT-3′). The siRNAs were transfected for 48 h using Lipofectamine 2000 following the manufacturer’s instructions.

### RNA isolation and quantitative real-time PCR

Total RNA was extracted from blood mononuclear cells or RAW 264.7 cells with TRIzol reagent following the manufacturer’s instructions. Reverse transcription was then performed using random hexamer primer and MMLV Reverse Transcriptase. qPCR for the quantitation of TRACP, MMP-9, Ctsk, and c-Src gene transcript was performed with 2× HotStart SYBR Green qPCR Master Mix using a ABI7500 real-time PCR instrument (Applied Biosystems, Thermo Fisher Scientific, Inc.). GAPDH was used as a control. The mRNA levels were calculated relative to internal control using 2^−ΔΔ*C*^_t_ method using real-time PCR system. The primer sequences are as below.

Human TRACP-F: 5′-ACACAGTGATGCTGTGTGGCAACTC-3′; human TRACP-R: 5′-CCAGAGGCTTCCACATATATGATGG-3′;

Human MMP-9-F: 5′-AGTTTGGTGTCGCGGAGCAC-3′; human MMP-9-R: 5′-TACATGAGCGCTTCCGGCAC-3′;

Human Ctsk-F: 5′-GGCCAACTCAAGAAGAAAAC-3′; human Ctsk-R: 5′-GTGCTTGCTTCCCTTCTGG-3;

Human c-Src-F: 5′-CCAGGCTGAGGAGTGGTACT-3′; human c-Src-R: 5′-CAGCTTGCGGATCTTGTAGT-3′;

Human GAPDH-F: 5′-AACTTTGGCATTGTGGAAGG-3′; human GAPDH-R: 5′-ACACATTGGGGGTAGGAACA-3′;

Mouse TRACP-F: 5′-CACTCCCACCCTGAGATTTGT-3′; mouse TRACP-R: 5′-CCCCAGAGACATGATGAAGTCA-3′;

Mouse MMP-9-F: 5′-GCAGAGGCATACTTGTACCG-3′; mouse MMP-9-R: 5′-TGATGTTATGATGGTCCCACTTG-3′;

Mouse Ctsk-F: 5′-CTCGGCGTTTAATTTGGGAGA-3′; mouse Ctsk-R: 5′-TCGAGAGGGAGGTATTCTGAGT-3;

Mouse c-Src-F: 5′-GAACCCGAGAGGGACCTTC-3′; mouse c-Src-R: 5′-GAGGCAGTAGGCACCTTTTGT-3′;

Mouse GAPDH-F: 5′-AGGTCGGTGTGAACGGATTTG-3′; mouse GAPDH-R: 5′-GGGGTCGTTGATGGCAACA-3′.

### Western blotting

Isolated blood mononuclear cells from healthy donors or patients with MM or RAW 264.7 cells after indicated treatment were homogenized in RIPA buffer with 0.01% protease and phosphatase inhibitors. The samples were then centrifuged at 12000×***g*** for 15 min at 4°C and the supernatants were collected. Protein lysates were separated by SDS/PAGE (10% gel) and transferred on to nitrocellulose membranes. Membranes were blocked with 5% non-fat milk in TBS containing 0.1% Tween-20 for 2 h at RT. Subsequently, membranes were incubated with relevant antibodies at 4°C overnight followed by horseradish peroxidase (HRP)–conjugated secondary antibody. Membranes were then incubated with ECL substrate and subjected to X-ray film exposure in the dark room. β-actin was used as a loading control. For the determination of p65 expression, the cytoplasm and nuclear proteins were isolated using Nuclear and Cytoplasmic Protein Extraction Kit.

### Statistical analysis

All data presented here were expressed as mean ± S.D. from at least three independent experiments. Data were analyzed by GraphPad Prism 6.0 (GraphPad Software Inc., San Diego, CA). Statistical differences were performed using unpaired two-tailed *t* test for comparison between two groups and one-way ANOVA followed by Tukey’s post hoc test for multiple comparison. The significance of difference was determined as indicated in the figure legends. A *P*<0.05 was considered statistically significant.

## Results

### I-BET151 dose-dependently suppresses osteoclast formation in isolated mononuclear cells from healthy donors and patients with MM

**Figure 1:**
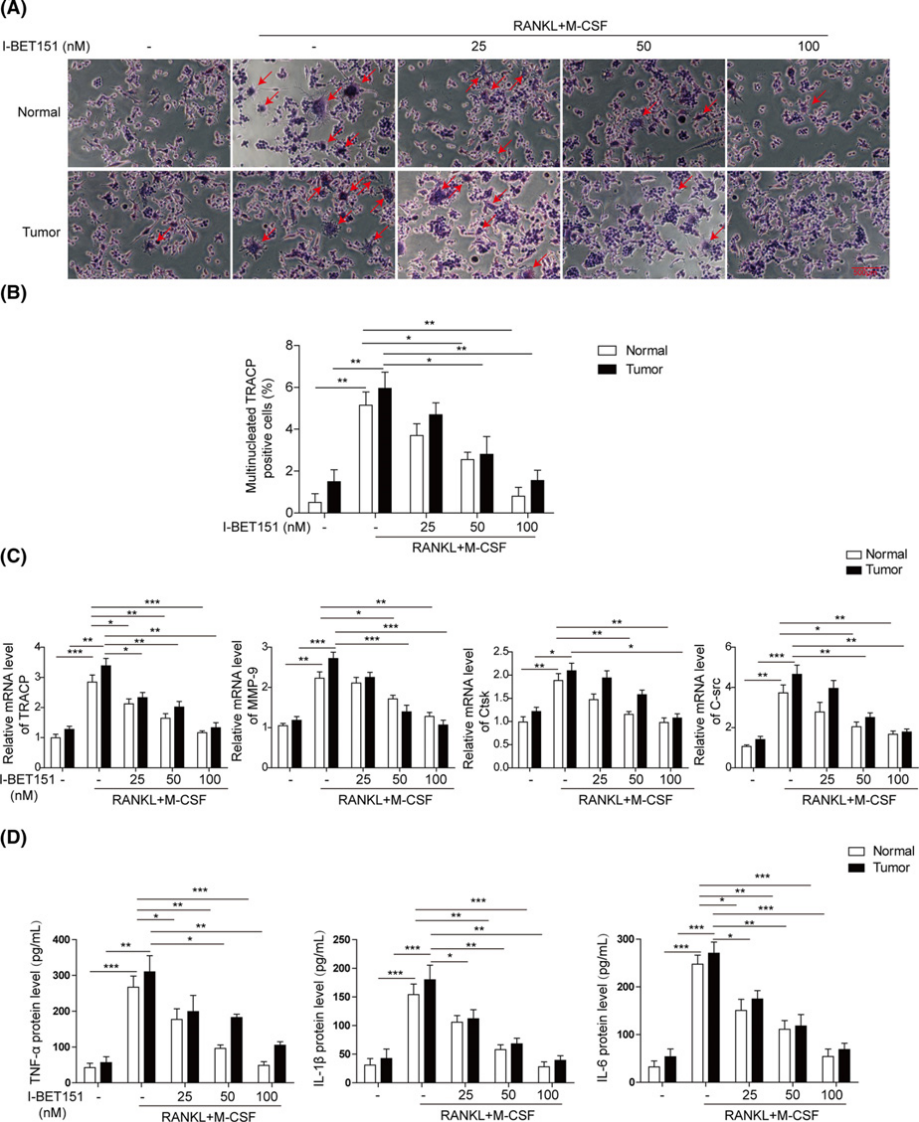
I-BET151 dose-dependently inhibited osteoclast differentiation of peripheral blood mononuclear cells from both normal and patients with MM (**A**) Representative images of TARCP staining showing osteoclast formation induced by the treatment RANKL and M-CSF in the presence or absence of I-BET151 at indicated concentrations in mononuclear cells from both normal and patients with MM. (**B**) The statistical analysis of images from (A) showing the percentage of multinucleated TRACP positive cells. (**C**) qPCR measurement of osteoclast-specific genes *TRACP, MMP-9, Ctsk*, and *C-Src* mRNA levels from the samples prepared as in (A). Data were normalized to the control. (**D**) ELISA determination of TNF-α, IL-1β, and IL-6 in the culture medium. The result was a representative of three independent experiments. Error bars represented mean ± S.D. *P*-values were determined by the one-way ANOVA followed by Tukey’s post hoc test; ***P<0.001, **P<0.01, *P<0.05.

Peripheral blood was collected from 20 pairs of healthy donors and patients with MM, selected in an age-matched manner. The isolated mononuclear cells were treated with induction factors RANKL and M-CSF in the absence or presence of I-BET151 at indicated concentrations. RANKL and M-CSF successfully stimulated the osteoclast formation and the percentage of multinucleated TRACP positive cells significantly up-regulated in both normal and patient samples (Figure 1A,B). However, co-treatment with I-BET151 dose-dependently reduced the number of multinucleated TRACP positive cells and osteoclast formation in both groups as revealed by the staining of osteoclast marker TRACP (Figure 1A,B). I-BET151 at 100 nM suppressed the number of multinucleated TRACP positive cells almost to the level as basal. Further, RANKL and M-CSF stimulated the expression of osteoclast-specific genes including TRACP, MMP-9, Ctsk and C-Src, which were down-regulated by the co-administration of I-BET151 in a dosage-dependent manner in both groups (Figure 1C). Then, the inflammatory cytokines in the culture medium were measured to investigate the effects of I-BET151 on cytokine secretion. I-BET151 significantly inhibited RANKL and M-CSF-induced promotion of TNF-α, IL-1β, and IL-6 secretion in a dose-dependent manner (Figure 1D). Moreover, the level of TRACP positive cells, osteoclast-specific gene expression, and inflammatory cytokine secretion were higher in tumor group than those in the normal group in most cases, indicating that the mononuclear cells from MM patients are easier to differentiate into osteoclasts. All these suggest that I-BET151 can suppress osteoclast formation and inflammatory cytokine secretion in MM.

### I-BET151 dose-dependently inhibits RANKL-NF-κB signal pathway in isolated mononuclear cells

**Figure 2:**
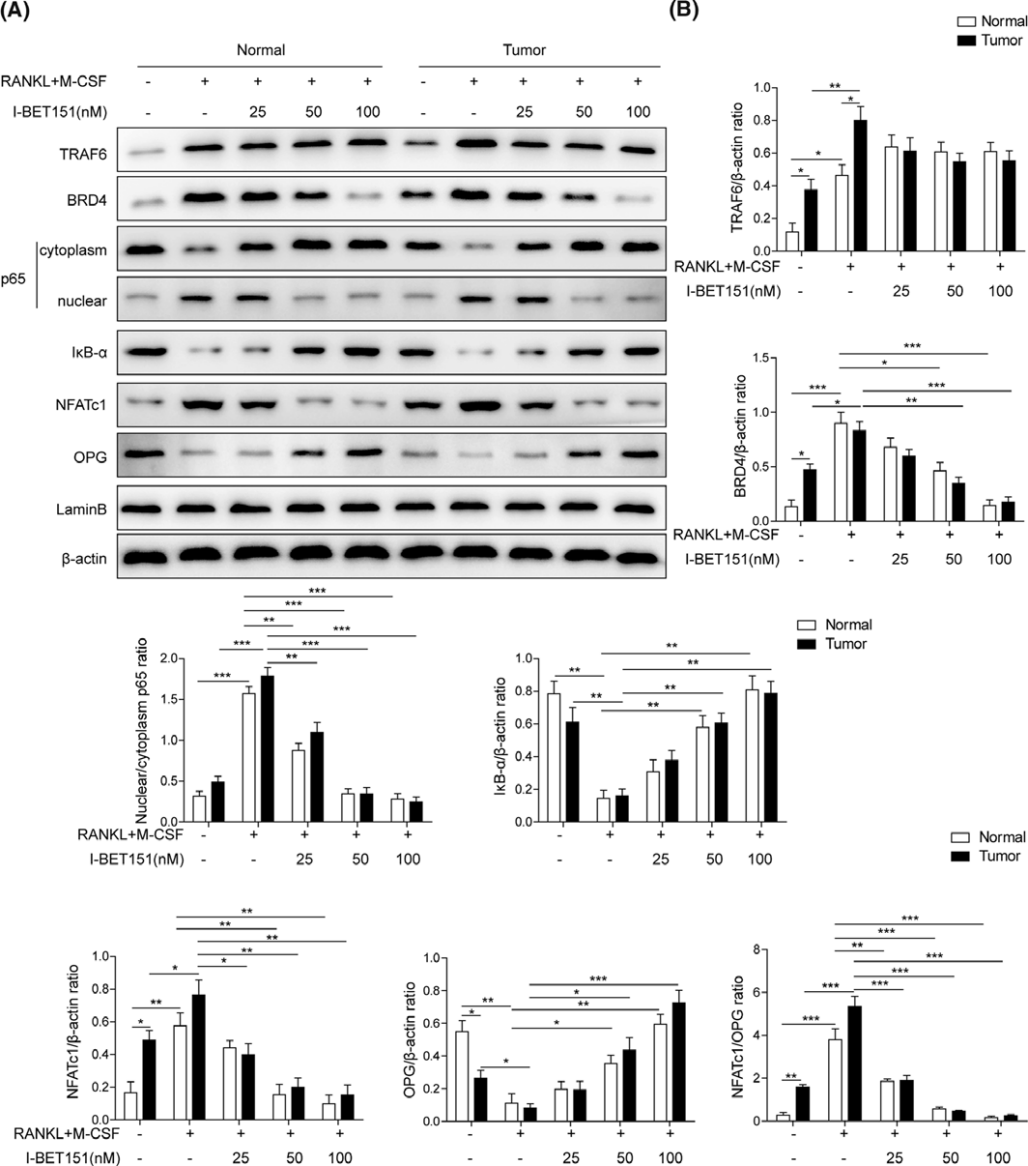
I-BET151 suppressed RANKL-NF-κB signal pathway in peripheral blood mononuclear cells (**A**) Representative images presenting the protein expression of TRAF6, BRD4, cytoplasm and nuclear p65, IκB-α, NFATc1, and OPG in response to the treatment with RANKL and M-CSF in the presence or absence of I-BET151 at indicated concentrations in peripheral blood mononuclear cells from both normal and patients with MM. β-actin was used as the loading control. The result was representative of three independent experiments. (**B**) The corresponding mean data were generated. Error bars represented mean ± S.D. *P*-values were determined by the one-way ANOVA followed by Tukey’s post hoc test; ***P<0.001, **P<0.01, *P<0.05.

RANKL is known to modulate osteoclast differentiation via the activation of NF-κB signal pathway [15,32]. Here, we consistently showed that the presence of RANKL and M-CSF markedly evoked the expressions of TRAF6, BRD4, and NFATc1, the degradation of IκB-α, the reduced cytosolic p65, and the increased nuclear p65 in blood mononuclear cells from either normal or tumor group, indicating RANKL and M-CSF induced the activation of RANKL-NF-κB signal pathway (Figure 2A,B). However, the effects on BRD4 and NFATc1 expression and NF-κB activation were significantly suppressed by the co-treatment with I-BET151 in a dose-dependent manner. TRAF6 is the upstream protein of BRD4 [33,34] and I-BET151 had no effect on TRAF6 expression. OPG, considered as the decoy receptor for RANKL, reduces excessive RANKL signaling [35,36]. Here, I-BET151 prevented the reduction in OPG level induced by RANKL and M-CSF in both normal and tumor groups. Additionally, the levels of TRAF6, BRD4, and NFATc1 expression, IκB-α degradation, and p65 nuclear translocation were higher in blood mononuclear cells from tumor samples than in those from normal group at basal levels. These results demonstrate that I-BET151 may suppress osteoclast differentiation via the inhibition of RANKL-NF-κB signal pathway.

### I-BET151 dose-dependently suppresses osteoclast formation in RAW 264.7 cells

**Figure 3:**
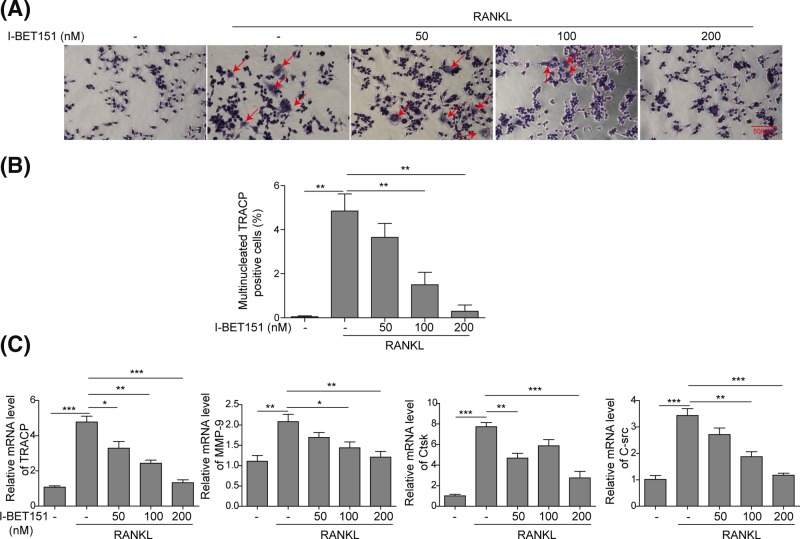
I-BET151 dose-dependently inhibited osteoclast differentiation in RAW 264.7 cells (**A**) Representative images of TARCP staining presenting osteoclast formation upon the challenge of RANKL in the presence or absence of I-BET151 at indicated concentrations in RAW 264.7 cells. (**B**) The statistical analysis of images from (A) showing the percentage of multinucleated TRACP positive cells. (**C**) The mRNA levels of TRACP, MMP-9, Ctsk, and C-Src were measured by qPCR. Data were normalized to the control. The result was representative of three independent experiments. Error bars represented mean ± S.D. *P*-values were determined by the one-way ANOVA followed by Tukey’s post hoc test; ***P<0.001, **P<0.01, *P<0.05.

We further investigated the molecular mechanisms by which I-BET151 inhibits osteoclast differentiation in RAW 264.7 cells. Treatment with RANKL induced 5% multinucleated TARCP positive cells while it was dose-dependently attenuated by the co-treatment with I-BET151 (Figure 3A,B). I-BET151 at 200 nM-decreased multinucleated TRACP positive cells was not significantly different from the basal. Moreover, the osteoclast-specific genes including *TRACP, MMP-9, Ctsk*, and *C-Src* were all stimulated by RANKL, and I-BET151 dose-dependently inhibited the expression of these specific genes at mRNA levels induced by RANKL (Figure 3C). These data suggest that I-BET151 dose-dependently inhibits osteoclast formation in RANKL-treated RAW 264.7 cells.

### BRD4 mediates I-BET151-induced prevention of osteoclast formation

**Figure 4:**
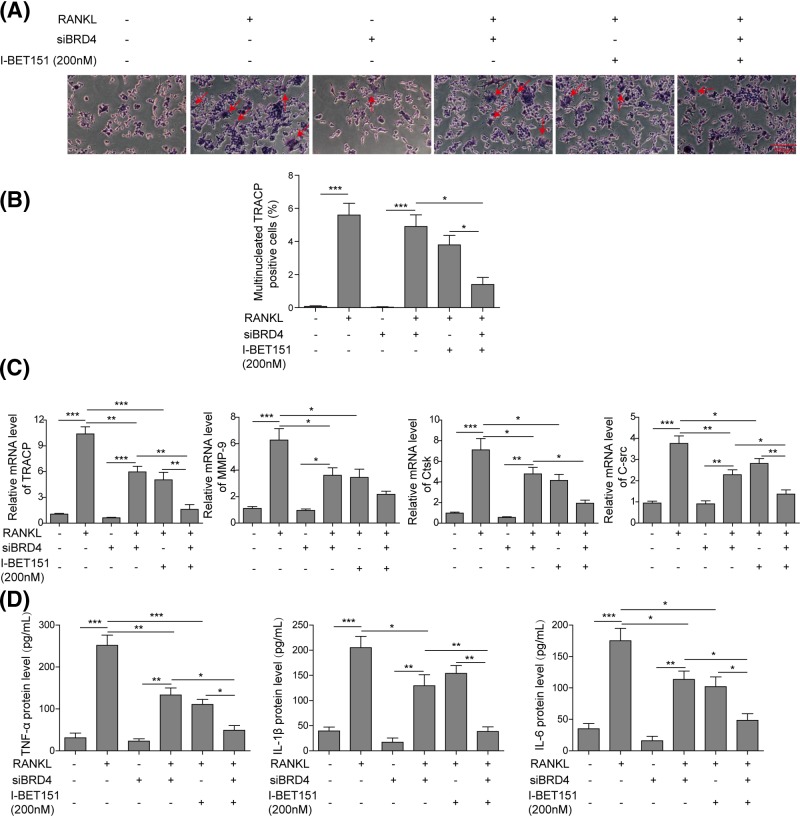
The knockdown of BRD4 promoted I-BET151-induced inhibition of osteoclast differentiation (**A**) Representative images of TARCP staining presenting osteoclast formation after RANKL treatment in the presence or absence of I-BET151 in RAW 264.7 cells with or without siBRD4 transfection. (**B**) The statistical analysis of images from (A) showing the percentage of multinucleated TRACP positive cells. (**C**) qPCR measurement of osteoclast-specific genes TRACP, MMP-9, Ctsk, and C-Src, from the samples prepared as in (A). Data were normalized to the control. (**D**) The levels of inflammatory cytokines TNF-α, IL-1β, and IL-6 in the culture medium were detected using ELISA. The result was representative of three independent experiments. Error bars represented mean ± S.D. *P*-values were determined by the one-way ANOVA followed by Tukey’s post hoc test; ***P<0.001, **P<0.01, *P<0.05.

To demonstrate the role of BRD4 in I-BET151-modulated inhibition of osteoclast formation, siRNA technique was applied. In RAW 264.7 cells, siBRD4 alone did not change osteoclast formation and the number of TRACP positive cells (Figure 4A,B), but siBRD4 suppressed RANKL-induced formation of multinucleated TRACP positive cells (Figure 4A,B). Moreover, the reduction in BRD4 expression promoted the effects of I-BET151 on the inhibition of osteoclast formation. Meanwhile, BRD4 knockdown showed little effect on the levels of TRACP, MMP-9, Ctsk, and C-Src, but significantly reduced RANKL-mediated stimulation of these osteoclast-specific genes (Figure 4C). Additionally, I-BET151-modulated inhibition of TRACP, MMP-9, Ctsk, and C-Src expression was enhanced by BRD4 knockdown (Figure 4C). Further, the levels of TNF-α, IL-1β, and IL-6 in the culture medium were not influenced by BRD4 knockdown (Figure 4D). BRD4 knockdown also suppressed RANKL-induced secretion of inflammatory cytokines and promoted the effects of I-BET151 on inhibition of cytokine secretion (Figure 4D). These indicate that I-BET151 prevents osteoclast formation and inflammatory cytokine secretion via targetting BRD4.

### BRD4 mediates I-BET151-induced suppression of RANKL-NF-κB signal pathway

**Figure 5:**
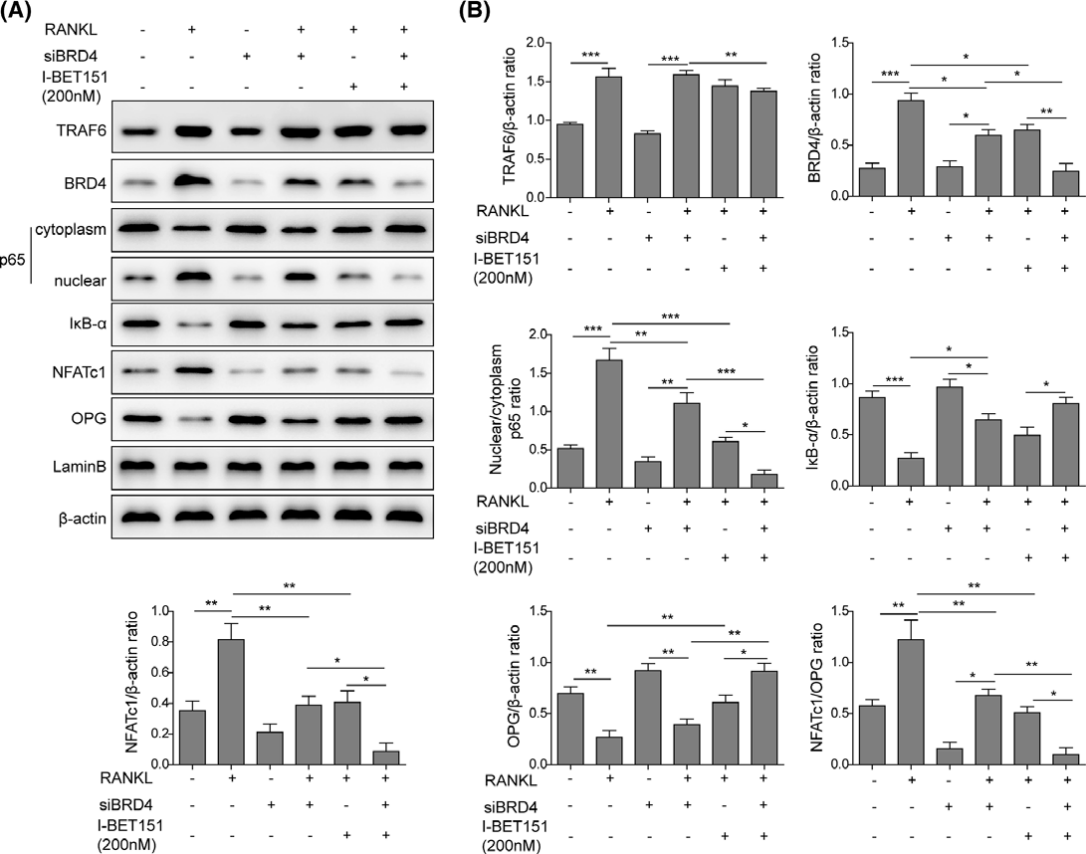
The knockdown of BRD4 suppressed RANKL-NF-κB signal pathway and strengthened the effects of I-BET151 (**A**) Representative images showing the protein expressions of TRAF6, BRD4, cytoplasm and nuclear p65, IκB-α, NFATc1, and OPG in response to the treatment with RANKL in the presence or absence of I-BET151 in RAW 265.7 cells with or without siBRD4 transfection. β-actin was used as the loading control. The result was representative of three independent experiments. (**B**) The corresponding mean data were generated. Error bars represented mean ± S.D. *P*-values were determined by the one-way ANOVA followed by Tukey’s post hoc test; ***P<0.001, **P<0.01, *P<0.05.

The effect of BRD4 on I-BET151-mediated RANKL-NF-κB signal pathway was further investigated. RANKL stimulated the expressions of TRAF6, BRD4, and NFATc1 (Figure 5). It also reduced cytosolic p65 level, promoted nuclear p65 expression, and attenuated the level of IκB-α in RAW 264.7 cells (Figure 5), indicating the nuclear translocation of p65 and the degradation of IκB-α. The expression of OPG was also inhibited by RNAKL. At basal condition, transfection with siBRD4 alone did not change the level of TRAF6 but reduced BRD4 and NFATc1 expression, inhibited p65 nuclear translocation and IκB-α degradation. BRD4 knockdown did not affect RANKL-induced expression of TRAF6 but prevented RANKL-induced expressions of BRD4 and NFATc1, nuclear translocation of p65, the degradation of IκB-α, and the down-regulation of OPG, which were all mimicked by I-BET151. Moreover, the effects of I-BET151 were promoted by BRD4 knockdown (Figure 5), suggesting that I-BET151 mediated inhibition of RNAKL-NF-κB signal pathway via targetting BRD4.

## Discussion

MM is a clonal disorder of plasma cells associated with increased secretion of inflammatory cytokines and the excessive formation of osteoclasts, leading to bone destruction and loss [37,38]. Owing to the essential effect of RANKL on osteoclastogenesis, RANKL has been a therapeutic target for the treatment of lytic bone diseases [39]. Both JQ1 and I-BET151 were small molecule inhibitors for BET proteins and JQ1 was demonstrated to attenuate inflammation and bone destruction in experimental periodontitis via neutralizing BRD4 enrichment at NF-κB, TNF-α, c-Fos, and NFATc1 [40]. A recent study reported that I-BET151 inhibited RANKL-induced osteoclastogenesis and bone loss in post-ovariectomy osteoporosis, inflammatory arthritis, and TNF-induced osteolysis mouse models by targetting MYC-NFAT axis [41]. Here we further presented that I-BET151 harbored the inhibitory effect on inflammatory cytokine secretion and osteoclast formation in MM, altogether demonstrating that the inhibition of BRD4 could be a promising strategy for the treatment of lytic bone disorders.

Previous studies have provided the preclinical evidence that BET inhibitors including I-BET151 and I-BET762 have considerable anti-myeloma activity both *in vitro* and *in vivo* [42,43]. The mechanistic study revealed that these inhibitors could induce cell cycle arrest and show anti-proliferative effects [42]. They also possessed pro-apoptotic effects against myeloid leukemia cells by suppressing the expression of pro-survival factor BCL-2 [42]. The current study presented that I-BET151 could dose-dependently inhibited the ratio of multinucleated TRACP positive cells and osteoclast differentiation, the expression of osteoclast-specific genes including TRACP, MMP-9, Ctsk, and C-Src, and the secretion of inflammatory cytokines including TNF-α, IL-1β, and IL-6 in peripheral blood from both healthy donors and patients with MM induced by RANKL and M-CSF. We further showed that mononuclear cells from MM patients were easier to differentiate into osteoclasts compared with those from the normal donors. These results provided further evidences for the beneficial effect of I-BET151 on osteoclast formation inhibition on MM.

NF-κB is a transcription factor that regulates the expression of genes involved in inflammation response, osteoclast formation, and controls cell death, cell proliferation, and differentiation [44–47]. BET protein regulates NF-κB activity by directly binding to its subunit RelA [48,49]. It was reported that BET inhibitor I-BET151 could suppress NF-κB activity and down-regulate NF-κB target genes for inflammation response, such as IL-6 and IL-8 [50]. Here we observed the inhibition effects on RANKL and M-CSF mediated the promotion of TRAF6 and NFATc1 expression, the induction of IκB degradation, and the increase in p65 nuclear translocation in response to I-BET151 treatment. This indicated that I-BET151 suppressed inflammatory cytokine secretion and osteoclast formation via the inhibition of RANKL-NF-κB signal pathway.

It was consistently observed that I-BET151 suppressed osteoclast formation, the mRNA level of osteoclast-specific genes, and inflammatory cytokine secretion in RAW 264.7 treated with RANKL, suggesting the successful establishment of cell line model for MM. The molecular mechanism by which I-BET151 regulates osteoclast formation and inflammatory cytokine secretion was then investigated in this cell line model. It was previously revealed that BRD4 regulated renal fibrosis by the modulation of NF-κB phosphorylation and acetylation [27]. And BRD4-activated NF-κB played the role in human kidney and lung carcinoma cells [25,51]. Here we further demonstrated that the knockdown of BRD4 suppressed osteoclast formation, the expression of osteoclast-specific genes, the secretion of inflammatory cytokines, and RANKL-NF-κB signal pathway activation, mimicking the effects of I-BET151. This suggests that BRD4-mediated NF-κB signal pathway inhibition may also be involved in I-BET151 effects on MM. Also, siBRD4 promoted the effects of I-BET151 on those events, suggesting the essential effects of BRD4 on I-BET151-mediated NF-κB signal pathway and osteoclast differentiation.

To sum up, the present study first revealed the inhibitory effect of BET inhibitor I-BET151 on osteoclast formation and inflammation response in MM via targetting BRD4-mediated RANKL-NF-κB signal pathway. This suggests that the inhibition of BRD4 could be a promising approach for the treatment of MM.

## Abbreviations

BET: bromodomain and extra-terminal domain
BRD: bromodomain-containing protein
Ctsk: cathepsin K
GAPDH: glyceraldehyde-3-phosphate dehydrogenase
IL: interleukin
M-CSF: macrophage colony stimulating factor
MM: multiple myeloma
MMP-9: matrix metalloproteinase-9
NFATc1: nuclear factor of activated T cells cytoplasmic
NF-κB: nuclear factor-κ-gene binding
OPG: osteoprotegerin
RANKL: receptor activator of NF-κB ligand
RIPA: radio-immunoprecipitation assay
RT: room temperature
TNF-α: tumor necrosis factor-α
TRACP: tartrate-resistant acid phosphatase
TRAF6: tumor necrosis factor receptor-associated factor 6
